# CROCUFID: A Cross-Cultural Food Image Database for Research on Food Elicited Affective Responses

**DOI:** 10.3389/fpsyg.2019.00058

**Published:** 2019-01-25

**Authors:** Alexander Toet, Daisuke Kaneko, Inge de Kruijf, Shota Ushiama, Martin G. van Schaik, Anne-Marie Brouwer, Victor Kallen, Jan B. F. van Erp

**Affiliations:** ^1^Perceptual and Cognitive Systems, Netherlands Organisation for Applied Scientific Research (TNO), Soesterberg, Netherlands; ^2^Kikkoman Europe R&D Laboratory B.V., Wageningen, Netherlands; ^3^Microbiology & Systems Biology, Netherlands Organisation for Applied Scientific Research (TNO), Zeist, Netherlands; ^4^Department of Research & Development Division, Kikkoman Corporation, Noda, Japan; ^5^Research Group Human Media Interaction, University of Twente, Enschede, Netherlands

**Keywords:** food pictures, food image database, valence, arousal, color, complexity, healthiness, desire to eat

## Abstract

We present CROCUFID: a CROss-CUltural Food Image Database that currently contains 840 images, including 479 food images with detailed metadata and 165 images of non-food items. The database includes images of sweet, savory, natural, and processed food from Western and Asian cuisines. To create sufficient variability in valence and arousal we included images of food with different degrees of appetitiveness (fresh, unfamiliar, molded or rotten, spoiled, and partly consumed). We used a standardized photographing protocol, resulting in high resolution images depicting all food items on a standard background (a white plate), seen from a fixed viewing (45°) angle. CROCUFID is freely available under the CC-By Attribution 4.0 International license and hosted on the OSF repository. The advantages of the CROCUFID database over other databases are its (1) free availability, (2) full coverage of the valence – arousal space, (3) use of standardized recording methods, (4) inclusion of multiple cuisines and unfamiliar foods, (5) availability of normative and demographic data, (6) high image quality and (7) capability to support future (e.g., virtual and augmented reality) applications. Individuals from the United Kingdom (*N* = 266), North-America (*N* = 275), and Japan (*N* = 264) provided normative ratings of valence, arousal, perceived healthiness, and desire-to-eat using visual analog scales (VAS). In addition, for each image we computed 17 characteristics that are known to influence affective observer responses (e.g., texture, regularity, complexity, and colorfulness). Significant differences between groups and significant correlations between image characteristics and normative ratings were in accordance with previous research, indicating the validity of CROCUFID. We expect that CROCUFID will facilitate comparability across studies and advance experimental research on the determinants of food-elicited emotions. We plan to extend CROCUFID in the future with images of food from a wide range of different cuisines and with non-food images (for applications in for instance neuro-physiological studies). We invite researchers from all parts of the world to contribute to this effort by creating similar image sets that can be linked to this collection, so that CROCUFID will grow into a truly multicultural food database.

## Introduction

Visual cues constitute a primary sensory input that allows predictions about the edibility and palatability of food. Through learning, visual food characteristics can become secondary reinforcers that affect eating-related behavior. The human brain has specific regions involved in the appetitive and affective processing of visual presentations of food stimuli (Killgore et al., 2003). The sight of food elicits a wide range of physiological, emotional and cognitive responses (van der Laan et al., 2011). Previous research has shown that viewing pictures of food not only activates the visual cortex, but also brain areas that code how food actually tastes (the insula/operculum) and the reward values of tasting it (the orbitofrontal cortex; Simmons et al., 2005). Food images are therefore typically considered as useful proxies for the real thing (e.g., Foroni et al., 2013; Blechert et al., 2014b; Miccoli et al., 2014, 2016). Food pictures are increasingly used as stimuli in research on the factors underlying affective and appetitive responses to foods (e.g., in online experiments Blechert et al., 2014b; Jensen et al., 2016), especially when the experimental paradigm limits actual consumption (e.g., experiments involving physiological measures such as EEG and fMRI; Killgore et al., 2003; Piqueras-Fiszman et al., 2014).

The sensory characteristics and evoked emotions of food are crucial factors in predicting a consumer’s food preference and therefore in developing new products (Dalenberg et al., 2014; Gutjar et al., 2015). Hedonic ratings alone do not predict food choice behavior accurately (Zandstra and El-Deredy, 2011; Griffioen-Roose et al., 2013). Emotions have an incremental predictive value over hedonic ratings alone, so that the best prediction can be obtained by combining both measures (Dalenberg et al., 2014; Samant and Seo, 2018). The relationship between food and emotions appears to be bidirectional: emotions influence eating behavior, while eating behavior also affects the consumer’s emotional state (Desmet and Schifferstein, 2008). Assessing emotional responses to foods could reveal product attributes that are a valuable source of information for product development and marketing going beyond traditional sensory and acceptability measurements (Thomson et al., 2010). Given the high failure rate of new food products in the market (between 60 and 80%: Köster and Mojet, 2007) and the fact that there is often only little difference in quality, price and design of different food products, knowledge about the affective responses of food products appears to be important for the food industry (Köster, 2003; Schifferstein et al., 2013). Therefore, it is important to obtain valid and reliable measurements of food-evoked emotions. Pictures are easy to present and have been widely used to investigate the different factors underlying affective responses in general (Lang et al., 1993) and appetitive responses to foods (Blechert et al., 2014a; Hebert et al., 2015). To facilitate multi-disciplinary and cross-cultural studies on food-related emotions we present a publicly available database containing images of a wide range of various foods from different cuisines, together with normative observer ratings.

Despite their recognized importance for research, only a few food image databases have been made publicly available to support research on human eating behavior (Chen et al., 2009; Foroni et al., 2013; Blechert et al., 2014b; Miccoli et al., 2014; Charbonnier et al., 2016) and they fall short on one or more of the following important criteria: (1) the public availability and ease of reproducibility, (2) the coverage of the full valence and arousal space, (3) the use of standardized recording methods, (4) the use of multiple cuisines and unfamiliar foods, (5) the availability of normative and demographic data (6) the image quality and capability for future applications. We will discuss the importance of these criteria in the following sections.

### Public Availability and Ease of Reproducibility

Researchers often select food images based on their personal preferences. As a result, the image sets are typically highly variable and difficult to compare, re-use or replicate (Pashler and Wagenmakers, 2012; Charbonnier et al., 2016). While some studies used their own set of images (Manzocco et al., 2013; Piqueras-Fiszman et al., 2014; Charbonnier et al., 2015), others collected stimuli from the internet (Foroni et al., 2013; Blechert et al., 2014b; Miccoli et al., 2014; Hebert et al., 2015). Also, most studies report little information about the used pictures and do not make the used image sets available to other researchers. It has been recognized that the comparability of research findings across studies using food pictures would benefit from the availability of public databases with standardized images (Foroni et al., 2013; Blechert et al., 2014b; Charbonnier et al., 2016).

### Coverage of the Affective Space

Existing food image databases typically focus on appetitive and positively valenced food pictures and lack aversive or negatively valenced ones. As a result, they cover only a limited part of the valence-arousal space, which significantly reduces their value for studies on food-evoked emotions. This is a result of the fact that food-related research has typically investigated positive and familiar food products while neglecting negative and unfamiliar ones, thus ignoring emotions and behaviors like disgust and rejection. This may suffice in product development and consumer research, but not in research on emotional responses to less liked and disliked food (Piqueras-Fiszman et al., 2014). Although positive food related emotional states are far more common, it is unavoidable that consumers sometimes experience negative food-related emotions (Desmet and Schifferstein, 2008; King et al., 2010; Cardello et al., 2012; Manzocco et al., 2013; Ng et al., 2013; Spinelli et al., 2015). Image databases intended to support research on human food-related behavior and to understand the full variety of emotional responses to food should therefore also include images of negatively valenced and unfamiliar products.

### Standardized Images

Standardization across image characteristics (e.g., background, color, brightness, contrast, size etc.) will enhance the reliability and validity of experimental results. However, most of the current databases contain unstandardized images of food items, sometimes simply collected from the internet and pasted on white backgrounds. The resulting large variation in image characteristics (e.g., contrast and brightness), the different angles at which foods are depicted, the variation in magnifications, the lack of a visual reference such as a plate together with the lack of shadows may compromise the reliability and validity of the experimental results from studies in which these images are used (Charbonnier et al., 2016).

### Multiple Cuisines and Unfamiliar Foods

Cultural background and familiarity are other factors that strongly influence food-related emotional responses (van Zyl and Meiselman, 2015) and determine food choice (Rozin, 1996). Databases should preferably include images representing a wide range of different food types (natural and processed) from a variety of cuisines. Existing food image databases include only a small range of (typically Western) cuisines, which limits their value for cross-cultural studies. Hence, there is a need for a food image database that includes food from different cuisines and has been validated across different nations, such as Latin-American, African, and Middle-Eastern.

### Normative and Demographic Data

The inclusion of different food types in a food image database enables the selection of food with different colors, caloric content, macronutrients, readiness to eat, flavor, nutritional composition, healthiness, and familiarity and the distinction of different classes such as vegetables, meat-containing dishes, fruits, and snacks (e.g., Blechert et al., 2014b).

A validated food-image database should preferably also include data on individual differences that are known to influence normative subjective ratings like age (Hoogeveen et al., 2015), gender (e.g., Cepeda-Benito et al., 2003; Toepel et al., 2012), body mass index (BMI; e.g., Berthoud and Zheng, 2012; Toepel et al., 2012), food preferences or diets (e.g., Stockburger et al., 2009) and psychophysiological state (e.g., Hoefling et al., 2009). Also, the measures included in the validation of the database should make a careful distinction between liking (the hedonic appraisal of food) and wanting (the motivation to consume: Berridge, 2004). In addition, the inclusion of images of artificial non-food objects (either related to food – like tableware or cutlery – or unrelated to food – like office supplies or toys), and organic non-food objects (like plants and flowers) is recommended to support brain studies (van der Laan et al., 2011).

### Image Quality and Suitability for Different Applications

Item-only (transparent) food images can be used in different experimental techniques like simulations of different ambient environments and multisensory cues. Recent studies have shown that the sensorial and hedonic experience of food is significantly influenced by the color (Genschow et al., 2012; Piqueras-Fiszman et al., 2012; Piqueras-Fiszman et al., 2013; Stewart and Goss, 2013; Michel et al., 2015b; Tu et al., 2016; Chen et al., 2018), shape (Piqueras-Fiszman et al., 2012; Stewart and Goss, 2013; Chen et al., 2018) and size (Van Ittersum and Wansink, 2012; Wansink and van Ittersum, 2013) of the plateware that is used for serving. In real-world settings, contextual factors such as ambience (Stroebele and De Castro, 2004), room color (Meléndez-Martínez et al., 2005; Oberfeld et al., 2009; Spence et al., 2014; Schifferstein et al., 2017), background sounds and music (McCarron and Tierney, 1989; Spence and Shankar, 2010; Crisinel et al., 2012; Fiegel et al., 2014), ambient temperature (Herman, 1993), the color and intensity of ambient lighting (Oberfeld et al., 2009; Suk et al., 2012; Hasenbeck et al., 2014; Spence et al., 2014; Cho et al., 2015) and ambient scents (Spence, 2015a) are also known to modulate the assessment and consumption of food and drinks (for reviews see Spence and Piqueras-Fiszman, 2014; Spence, 2018). It has been suggested that these effects reflect a transfer of sensations through cross-modal correspondences (Spence, 2011). However, the exact nature of these effects and the way in which they interact is still a topic of research. Acquiring further knowledge about the way in which multisensory contextual and ambient cues interact and affect human food related behavior will be of great value in retail and restaurant settings and may help to improve food experience and consumption behavior (e.g., to fight obesity, to enhance the consumption experience of elderly or people in hospital and care facilities, etc.). Virtual Reality (VR: Gorini et al., 2010; Nordbo et al., 2015; Ung et al., 2018), Augmented Reality (AR: Narumi et al., 2012; Pallavicini et al., 2016), Mixed Reality (MR: Huisman et al., 2016) or other immersive technologies (Bangcuyo et al., 2015; Liu et al., 2018) appear to be promising tools for this kind of research (see also Spence and Piqueras-Fiszman, 2014). The extension of VR and AR systems with novel multisensory (taste, smell, and tactile) interfaces can further enhance the perceived reality of food imagery (Braun et al., 2016) and will provide researchers control over the various inputs that determine a given food experience (Velasco et al., 2018). This may significantly increase the effectiveness of these systems for studies on food-related emotions and behavior, personal health and wellbeing (Comber et al., 2014; Obrist et al., 2016). Multisensory HCI systems may for instance be used to match the visual, auditory and olfactory characteristics of a simulated table or restaurant setting to food (e.g., Reinoso Carvalho et al., 2016). Transparent item-only images can be overlaid on images of plates with different shapes, colors and textures to study the effects of the visual characteristics of the plate on the appraisal of food. To study the effects of the visual characteristics of the environment, these plated food images can in turn be overlaid on images, movies or VR renderings of different backgrounds (e.g., images showing plated food placed on tables covered with different tablecloths or on different natural surfaces, movies showing environments with different lighting characteristics, etc.). To study the effects of ambient sound, (dynamic) lighting or social presence, the plated food images can be overlaid on movies showing dynamic environments with different ambient characteristics. Adding smells, tastes or tactile stimulation to the image presentation may serve to further enhance the realism of this type of studies (Velasco et al., 2018). Using item-only images in augmented reality settings will provide an efficient way to study the effects of ambient characteristics on human response to many different types of food (Nishizawa et al., 2016).

### The CROCUFID Approach

To complement the currently available food image databases and to further support systematic neuroscientific and behavioral studies on the emotional impact of food we present CROCUFID: a CROss-CUltural Food Image Database with high-resolution images of different types of food from various (currently mainly Western and Asian) cuisines, together with normative ratings (by participants from the United Kingdom, North-America, and Japan) of valence, arousal, desire-to-eat, perceived healthiness, and familiarity, complemented with computational measures of physical food properties that are known (or *a priori* considered likely) to influence food experience. To make it useful for brain (e.g., fMRI) studies, the dataset also contains images of non-food objects. To cover the full valence-arousal space, CROCUFID includes images of food with different degrees of appetitiveness (fresh, unfamiliar, molded or rotten, contaminated, and partly consumed). The inclusion of food types from different (Western and Asian) cuisines allows the images to be used in a culturally diverse population. The images resemble the viewing of a plate of food on a table during meal time and will therefore be a useful tool to assess emotional responses in studies that limit actual consumption (e.g., experiments involving physiological measures such as EEG and fMRI). To afford the use of CROCUFID in human–computer interaction (HCI) studies, all images in the dataset are also provided as item-only (transparent PNG) images. CROCUFID complements the F4H image collection since both were registered using the same photographing protocol (Charbonnier et al., 2016).

## Related Work

In this section we first describe the characteristics of some currently and publicly available food image databases that have been designed to support neuroscientific and behavioral research on human eating behavior and preferences. Then we review the databases that have been constructed to develop and train automatic food recognition and ingredient or recipe retrieval algorithms. To the best of our knowledge, these databases are currently the only ones publicly available that contain images of a wide range of different cuisines. However, they generally appear to be unsuitable for systematic research on human food-related behavior since they typically contain real-life images with largely varying backgrounds, taken from different points of view, with different scales and rotation angles and under varying lighting conditions. Next, we describe the value of CROCUFID for studies on the effects of environmental characteristics and background context on human food experience. Finally, we discuss how CROCUFID can be used to perform cross-cultural food studies.

### Food Image Databases for Human Observer Studies

Table 1 provides an overview of publicly available food image databases for human observer studies.

**Table 1:** Overview of food image databases for human observer studies.

Databases	Coverage of affective space	Recording methods	Cuisines	Availability of normative (and demographic) data	Remarks
FRIDa (Foroni et al., 2013)	Mainly positive valence	Not standardized	Mainly Western	Valence, Arousal, Familiarity (Italian)	• Low resolution (530 pixels × 530 pixels)
					• Collected from Internet
					• Includes non-food images
Food-Pics (Blechert et al., 2014b)	Mainly positive valence	Not standardized	Mainly Western	Valence, Arousal, Familiarity, Recognizability, Complexity, Palatability (German and North American)	• Low resolution (600 pixels × 450 pixels)
					• Collected from Internet No fixed background
OLAF (Miccoli et al., 2014, 2016)	Mainly positive valence	Not standardized	Mainly Western	Valence, Arousal, Dominance, Food Craving (Spanish)	• High resolution (4000 pixels × 3000 pixels)
					• Includes some low valence images from IAPS
					• Includes non-food images
F4H (Charbonnier et al., 2016)	Mainly positive valence	Standardized	Mainly Western	Liking, Healthiness, Recognizability, Perceived Calories (Greek, Dutch, Scottish, German, Hungary, and Swedish)	• Resolution (3872 pixels × 2592 pixels)
					• All images registered by the authors
					• Includes non-food images


The Foodcast Research Image Database (FRIDa^1^: Foroni et al., 2013) contains images of predominantly Western natural, transformed, and rotten food, natural and artificial non-food objects, animals, flowers and scenes, along with a description of several physical product properties (e.g., size, brightness and spatial frequency content) and normative ratings (by Italian participants) on several dimensions (including valence, arousal, and familiarity). The items were collected from the internet, pasted on a white background and have a low resolution (530 pixels × 530 pixels).

The Food-pics database^2^ (Blechert et al., 2014b) contains images of predominantly Western food types, together with normative ratings (by participants from German speaking countries and North America) on familiarity, recognizability, complexity, valence, arousal, palatability, and desire to eat. The items were collected from the internet, pasted on a white background and have a low resolution (600 pixels × 450 pixels). Food-pics has been designed to support experimental research on food perception and eating behavior in general.

The Open Library of Affective Foods (OLAF^3^: Miccoli et al., 2014, 2016) is a database of food pictures representing four different types of Western food (vegetables, fruit, sweet and salty high-fat foods), along with normative ratings (by Spanish students) of valence, arousal, dominance and food craving. The images have a high resolution (up to 4000 pixels × 3000 pixels) and include food served in restaurants and homemade meals, and display non-food items in the background to increase their ecological value and to resemble the appearance of images from the International Affective Picture System (IAPS: Lang et al., 2005). The four selected food categories focus on the extremes of the low-calorie/high-calorie food axis. Although OLAF was specifically compiled to be used in studies on the affective and appetitive effects of food, it contains no food images with negative valence. To remedy the lack of negative valence images and to provide affective anchors, OLAF was extended with 36 non-food images from the IAPS (12 from each of the three valence categories pleasant, neutral, unpleasant) that cover the full valence-arousal space.

The Full4Health Image Collection (F4H^4^) contains 228 images of Western food types of different caloric content, together with normative ratings (by adults from Greece, Netherlands and Scotland, and by children from Germany, Hungary, and Sweden) on recognizability, liking, healthiness and perceived number of calories. In addition, F4H also includes images of 73 non-food items. The images have a high resolution (3872 pixels × 2592 pixels) and were registered according to a standardized photographing protocol (Charbonnier et al., 2016). F4H has been designed for health-related studies in which (perceived) caloric content is of interest and contains no food pictures with negative valence.

### Food Image Databases for Automatic Recognition Studies

Table 2 provides an overview of publicly available food image databases for automatic image recognition studies.

**Table 2:** Overview of food image databases for autonomic recognition studies.

Databases	Coverage of affective space	Recording methods	Cuisines	Availability of normative (and demographic) data	Remarks
PFID (Chen et al., 2009)	A small part of valence and arousal space	Not standardized	Mainly Western	Not available	• High resolution (2592 × 1944 pixels)
					• Collected by the authors
					• No fixed background
NU FOOD (Takahashi et al., 2017)	Mainly positive valence	Standardized	Some Asian, Some Western	Not available	• Only 10 different cuisines (six Asian and four Western cuisines)
					• No resolution specified
ChineseFoodNet (Chen et al., 2017)	Mainly positive valence	Not standardized	Only Chinese	Not available	• Variable resolution
					• Collected from Internet (185,628 images)
					• No fixed background
UNICT Food Dataset 889 (Farinella et al., 2015)	Mainly positive valence	Not standardized	Italian, English, Thailand, Indian, Japanese etc.	Not available	• Variable resolution
					• Collected with smartphones (3,583 images)
					• No fixed background
UEC-Food 100 (Matsuda et al., 2012) UEC-Food 256 (Kawano and Yanai, 2015)	Mainly positive valence	Not standardized	France, Italy, United States, China, Thailand, Vietnam, Japan, Indonesia, etc.	Not available	• Variable resolution
					• Collected from Internet
					• No fixed background
UPMCFOOD-101 (Wang et al., 2015) ETHZFOOD-101 (Bossard et al., 2014)	Mainly positive valence	Not standardized	More than 101 international food categories	Not available	• Variable resolution
					• Collected from Internet
					• No fixed background
VIREO-172 (Chen and Ngo, 2016)	Mainly positive valence	Not standardized	Only Chinese	Not available	• Variable resolution
					• Collected from Internet (110,241 images)
					• No fixed background


The Pittsburgh Fast-Food Image Dataset (PFID^5^: Chen et al., 2009) contains still images, stereo pairs, 360-degrees videos and videos of Western fast-food and eating events, acquired in both restaurant environments and laboratory settings. The dataset represents 101 different foods with information on caloric content and is primarily intended for research on automated visual food recognition for dietary assessment. Although the images are registered at a high resolution (2592 pixels × 1944 pixels), the products that are shown occupy only a small region of the image, while the luminance, structure and shadowing of the background vary largely across the image set (due to undulations in the gray cloth in the background). Since the database only contains images of fast-food, it covers only a small part of the valence-arousal space and is therefore not suitable for systematically studying the emotional impact of food. Also, the database is significantly (Western) cultural specific, implying the previously mentioned cross-cultural restrictions for this dataset as well.

The NU FOOD 360×10 database^6^ (Takahashi et al., 2017) is a small food image database containing images of 10 different types of food, each shot at three elevation angles (30, 60, and 90 degrees) from 12 different angles (30 degree spacing). Six of the 10 foods are typically Asian (Sashimi, Curry and rice, Eel rice-bowl, Tempura rice-bowl, Fried pork rice-bowl, and Tuna rice-bowl), while the remaining four represent Western food (Beef stew, Hamburger steak, Cheese burger, and Fish burger). The food categories were selected considering the variation of the appearance in both color and shape. However, for reasons of convenience and reproducibility plastic food samples were used instead of real ones. This may degrade the perceived naturalness of the images.

The ChineseFoodNet^7^ (Chen et al., 2017) contains over 185,628 images of 208 Chinese food categories. The images in this database are collected through the internet and taken in real world under unconstrained conditions. The database is intended for the development and training of automatic food recognition algorithms.

The UNICT Food Dataset 889 (UNICT-FD889^8^, Farinella et al., 2015) contains 3,583 images of 889 distinct real meals of different nationalities (e.g., Italian, English, Thai, Indian, Japanese, etc.). The images are acquired with smartphones both with and without flash. Although it is an extended, cross-cultural database, images are not standardized and provided with emotional scores of, e.g., valence and arousal. Additionally the technical quality of the presented images consequently fluctuates. This is most likely by design as the database is intended for the development of automatic image retrieval algorithms.

The UEC-Food100 (Matsuda et al., 2012) and UEC-Food256^9^ (Kawano and Yanai, 2015) are both Japanese food image datasets, containing 100 and 256 food categories, respectively, from various countries such as France, Italy, United States, China, Thailand, Vietnam, Japan, and Indonesia. The dataset was compiled to develop algorithms that automatically retrieve food images from the internet. The images have widely varying backgrounds (e.g., different compositions and lighting of plates etc.), implying that this has limited value for human neurophysiological food-related studies.

The UPMCFOOD-101 (Wang et al., 2015) and ETHZFOOD-101^10^ (Bossard et al., 2014) datasets are twin datasets with the same 101 international food categories but different real-world images, all collected through the internet. The images of UPMCFOOD-101 are annotated with recipe information, and the images of ETHZ-FOOD-101 are selfies. The datasets were compiled to develop automatic systems for recipe recognition, an exercise that requires significantly other pictorial features than applications that intent to evoke discriminative, though well-defined, emotional responses.

VIREO-172^11^ (Chen and Ngo, 2016) is a dataset containing 110,241 images of popular Chinese dishes from 172 categories, annotated with 353 ingredient labels. The images are retrieved from the internet and have widely varying backgrounds, implying the associated diversity in technical quality. Like some previously mentioned databases, this database is intended to develop automatic cooking recipe retrieval algorithms with ingredient recognition.

## The Crocufid Database

### Recording Material and Protocol

The CROCUFID images were created following the photographing protocol presented by Charbonnier et al. (2016), as shown in Figure 1. The images were taken with a Canon EOS 1300D high-resolution digital single lens mirror reflex camera that was mounted on a tripod and stored both in RAW (CR2) and JPEG format. The focal length used was 34.0 mm, the shutter speed was 1/50 s and the aperture value was 4.5 for each picture. In two pictures (image number 20 and 88) a blue plate was used to enable the study of color contrast effects (we plan to extend the database with more pictures of food arranged on plates with different colors in the future). Consequently, all other (food and non-food) items were placed on a white IKEA plate (type FÄRGRIK^12^) with a diameter of 27 cm. The plate itself was placed in a 38 cm × 38 cm × 38 cm Foldio2 photo studio (a cubic photo tent made from white plastic sheets that soften and reflect the light from two LED strips above and below^13^). The camera was placed on a tripod. The Foldio2 studio and the camera were both rigidly attached to a common baseplate. The optical center of the camera was 39.5 cm from the center of the Foldio2 studio and 38 cm above the base plane. The viewing angle of the lens was approximately 45° downward, which resembles the viewing of a plate of food on a table during mealtime. A thin (5 mm thickness) gray foam board with a circular hole with the same diameter as the base of the IKEA plate was placed inside the Foldio2 studio, to ensure that the plate could easily be replaced in the same position for each image registration. The placement of the plate was checked by projecting a preregistered reference image of an empty plate over the actual plate using the ‘Show Overlay’ function of the camera. The foam board had a light gray color to ensure sufficient luminance contrast of the plate with its background, which enables automatic digital background correction (e.g., using Adobe Photoshop or Matlab). At the start of each food-registration session a picture of an X-Rite ColorChecker Passport^14^ was made to enable post-registration white-balance correction and reproduction of the original colors in each image (using Adobe Photoshop or the accompanying ColorChecker Camera Calibration software). The images were partly registered in the Netherlands (*N* = 426; images 1–357 and 565–633) and partly in Japan (*N* = 414; images 358–564 and 634–840).

**FIGURE 1:**
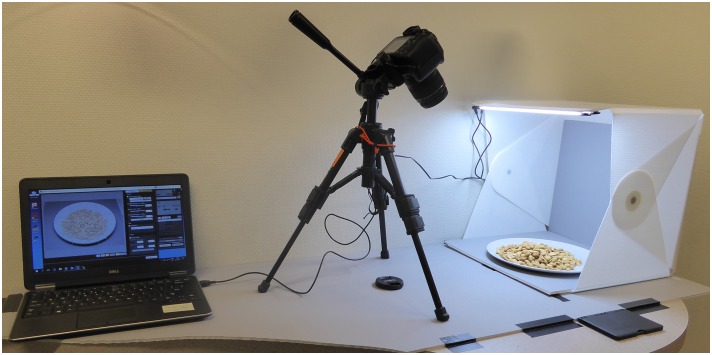
Standardized photographing protocol set-up (see: Charbonnier et al., 2016). A laptop **(left)** was used to control the camera **(middle)** settings and take the pictures of the plate with food in the Foldio2 photo studio **(right)**.

The plate’s background was standardized in all images as follows. First, a binary mask image was created in Photoshop by segmenting the reference image of the empty plate into a plate and background area followed by thresholding. Then, this mask image was used in Matlab 2018b^15^ to segment the plate from each of the 840 images and combine it with the segmented background from the reference image. Some smoothing was applied to the edges of the plate to prevent abrupt (noticeable) luminance transitions. In addition, the luminance of the (visible part of the) IKEA plate was equalized throughout the image set.

**FIGURE 2:**
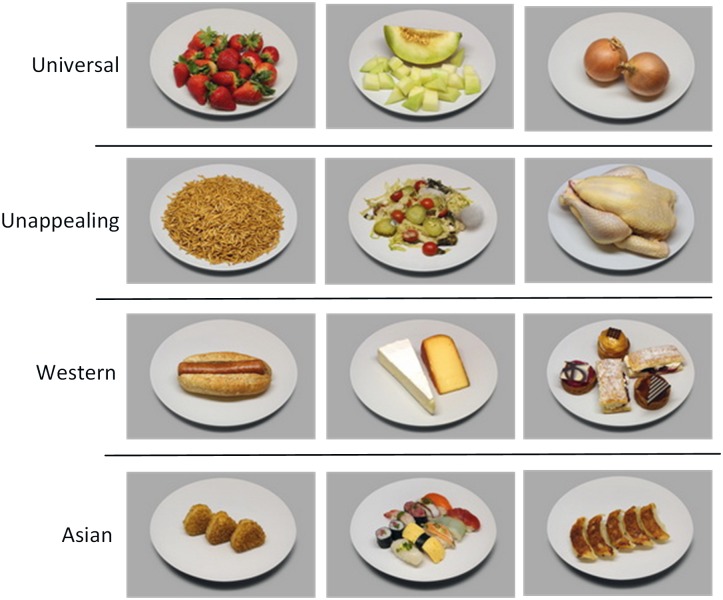
Representative images of four typical food categories (Universal, Unappealing, Western, Asian).

**FIGURE 3:**
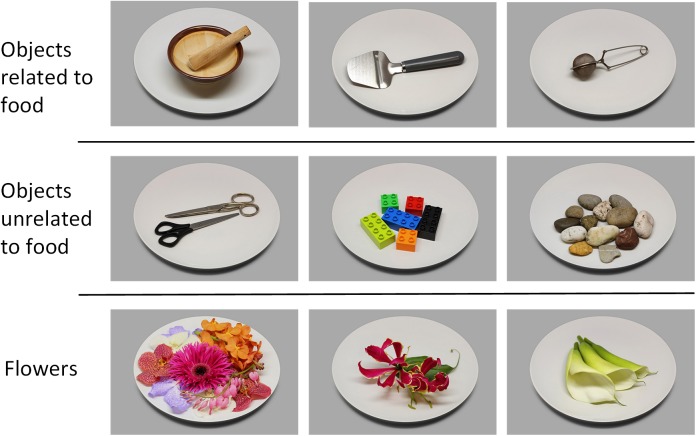
Representative images of three typical non-food categories (objects related or unrelated to food, flowers).

**FIGURE 4:**
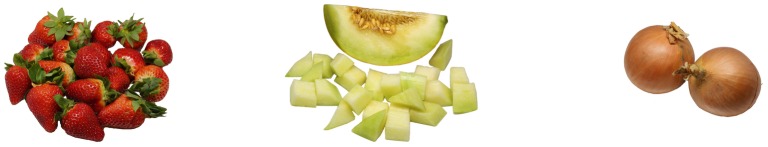
Some examples of item-only food images.

The CROCUFID database currently comprises 840 images: 675 food pictures (for some examples see Figure 2) and 165 non-food pictures (Figure 3). Detailed metadata are provided for the first 479 food images. All images in CROCUFID are high-quality standardized 8 bits color pictures with a resolution of 5184 pixels × 3456 pixels. For the validation studies reported in this paper the images were reduced in size to 1037 pixels × 691 pixels. A corresponding set of item-only (food or non-food) images representing the displayed items on a transparent background (for some examples see Figure 4) was created by using the PhotoScissors background removal tool^16^ for the initial separation of the food from the background, followed by the magic wand and color range selection tools in Adobe Photoshop to remove small remaining background sections inside the convex hull of the food area. These transparent item-only images can for instance be used to study the effects of background characteristics (e.g., plate size, color, texture, etc., see e.g., Piqueras-Fiszman et al., 2012, 2013; Van Ittersum and Wansink, 2012; Stewart and Goss, 2013) and plating arrangements (e.g., centered on a plate or off-center: Michel et al., 2015a; Velasco et al., 2016) by simply superimposing them onto images of different plates or even video clips of dynamic background textures (as in Toet, 2016). After thresholding and binarization the item-only PNG images can also serve as masks to restrict the calculation of computational measures to the area of the item on the plate.

### Image Features

Objective features like visual texture (Lucassen et al., 2011), complexity (Forsythe et al., 2011; Marin and Leder, 2013) and colorfulness (Cano et al., 2009) influence affective observer responses (measures of valence and arousal) to visual scenes in general (e.g., natural textures: Toet et al., 2012). Since our first sensory contact with food is typically through our eyes (van der Laan et al., 2011; Spence et al., 2015) it is not surprising that these visual cues also affect our responses to food (Zellner et al., 2010; Wadhera and Capaldi-Phillips, 2014). It has for instance been found that our appraisal and consumption of food depends on visual impressions like perceived regularity or randomness (Zellner et al., 2011; Zampollo et al., 2012), complexity (Mielby et al., 2012), spatial layout (Zampollo et al., 2012; Michel et al., 2015a; Szocs and Lefebvre, 2017), area of the plate covered (Van Ittersum and Wansink, 2012), and color content (Zellner et al., 2010; Zampollo et al., 2012; Piqueras-Fiszman and Spence, 2014; Spence, 2015b; Foroni et al., 2016; König and Renner, 2018). We therefore complemented the dataset with several computational measures that evaluate visual features (derived from the image luminance and chrominance distribution) that are known (or *a priori* likely) to influence affective image appraisal. These measures allow the user to select CROCUFID images based on their physical properties.

The first seven measures characterize the perceived image texture (i.e., the low-level spatial arrangement of color or intensities in an image). Five of these measures are only defined for grayscale images. To enable the computation of these measures the color images were therefore first converted to grayscale images with the MATLAB *rgb2gray* function before calculating these texture measures.

*Entropy* (S) is a statistical measure that characterizes the degree of randomness of the input image texture: entropy is 0 if all pixels have the same intensity value. Entropy was calculated with the standard MATLAB function *entropy*.

*Power* (P) is the average of the power spectral density over the image support (in decibels), computed from the discrete 2D Fourier transform of the image. This measure reflects the mean variations in image intensity.

The remaining three grayscale image texture measures (contrast, energy, and homogeneity) were computed from the Gray Level Co-occurrence Matrix (GLCM, which is a classic technique used for image texture analysis and classification: Haralick et al., 1976) using the MATLAB function *graycoprops*.

*Contrast* (C) measures the intensity contrast between a pixel and its neighbors averaged over the whole image and is equal to 0 for a constant valued image (Corchs et al., 2016).

*Energy* (E) is the sum of squared elements in the GLCM and is equal to 1 for a constant valued image (Corchs et al., 2016).

*Homogeneity* (H) measures the closeness of the distribution of elements in the GLCM with respect to the GLCM diagonal and is equal to 1 for a diagonal GLCM (Corchs et al., 2016).

We also computed two measures that describe the texture of color images. These measures are based on the Pyramid Histogram of Oriented Gradients (PHOG) image representation that was originally developed for object recognition and classification (Bosch et al., 2007) and have been used to characterize the aesthetics and liking of images and artworks (Redies et al., 2012; Braun et al., 2013; Hayn-Leichsenring et al., 2017). The PHOG descriptors are global feature vectors based on a pyramidal subdivision of an image into sub-images, for which Histograms of Oriented Gradients (HOG: Dalal and Triggs, 2005) are computed.

*Self-similarity* (SS) is computed using the Histogram Intersection Kernel (HIK: Barla et al., 2002) to determine the similarity between Hog features at the individual levels of the PHOG (for details see Redies et al., 2012; Braun et al., 2013). Images of natural (growth) patterns typically have a highly self-similar (fractal) structure, whereas artificial (man-made) structures typically have a low self-similarity.

*Anisotropy* (AN) describes how the gradient strength varies across the orientations in an image. Low anisotropy means that the strengths of the orientations are uniform across orientations and high anisotropy means that orientations differ in their overall prominence (Redies et al., 2012).

The next six measures quantify the structural image complexity. The complexity of an image depends on the number of its structural components, their heterogeneity, (e.g., a single shape repeated vs. multiple distinct shapes), their regularity (e.g., simple polygons vs. more abstract shapes) and the regularity of the arrangement of elements (e.g., symmetry, distribution characteristics; see Figure 1 of Berlyne, 1958).

The *Compression Ratio* (CR) between the original (uncompressed) and (JPEG or GIF format) compressed file sizes is a computational measure that is positively correlated with ratings of subjective image complexity (Marin and Leder, 2013). The file size of a digitized image is a measure of its structural information content (Donderi, 2006). Compression algorithms use image redundancy or predictability to reduce the file size, such that more complex (or less predictable) images need more elements. Using the lossless JPEG compression mode of the MATLAB *imwrite* function we computed the JPEG based compression ratio (CRjpeg) which has been shown to be a reliable measure of subjective complexity across various image domains (Marin and Leder, 2013). We also computed the GIF based compression ratio (CRgif) using Adobe Photoshop (settings: palette local selective, colors 256, forced black-white colors, no transparency, dither diffusion 75%, exact colors and normal order of lines, see: Marin and Leder, 2013).

*Feature Congestion* (FC) is a visual clutter measure that implicitly captures the notion of spatial disorder by computing a weighted average of the local feature (color, orientation, and luminance) contrast covariance over multiple (typically three) spatial scales (Rosenholtz et al., 2007). Larger FC values correspond to higher levels of visual clutter. The FC measure was calculated using the MATLAB code provided^17^ by Rosenholtz et al. (2007).

*Subband Entropy* (SE) is a clutter measure that encodes the image information content (or redundancy) by computing a weighted sum of the entropies of the luminance and chrominance image subbands (Rosenholtz et al., 2007). Larger SE values correspond to higher levels of visual clutter. The SE measure was calculated using the MATLAB code provided^18^ by Rosenholtz et al. (2007).

The *Number of Proto-Objects* (NPO) is the number of image segments or super-pixels with similar intensity, color and gradient orientation features (the proto-objects: Yu et al., 2014). Larger NPO values correspond to higher levels of visual clutter. The NPO measure was calculated using the MATLAB code provided by the authors^19^.

The *Mean Information Gain* (MIG) is defined as the difference between the spatial heterogeneity (i.e., the joint entropy among neighboring pixels) and the non-spatial heterogeneity (i.e., the probability of observing a pixel value independently of its location in the image) of an image (Andrienko et al., 2000; Proulx and Parrott, 2008). The MIG does not require knowledge of the ‘maximal’ entropy of the image (which is sometimes hard to compute or even to define), and accounts for the inherent spatial correlations. The MIG increases monotonously with spatial randomness and ranges over 0–1: MIG = 0 for uniform patterns and MIG = 1 for random patterns. The MIG index is a well-known complexity measure in statistical physics (Wackerbauer et al., 1994) and has successfully been used to quantify the complexity of two-dimensional patterns (Andrienko et al., 2000) and ecological habitats (Proulx and Parrott, 2008). The images were transformed to HSV format and the pixel value range was normalized from 0 to 10 before calculating MIG values independently for the color (Hue: MIGh), chroma (Saturation: MIGs), and intensity (Value: MIGv) components of each image. The MIG measure was calculated using the MATLAB code provided by Proulx and Parrott (2008) at http://complexity.ok.ubc.ca/projects/measuring-complexity.

The *Mean Gradient Strength* (MGS) or mean edge strength is a valid measure of subjective image complexity (Braun et al., 2013) and is based on the observation that the subjectively perceived level of image complexity increases with its number of edges. To compute the MGS we first transformed the images from RGB to Lab format (using the MATLAB function *rgb2lab*). Then the MATLAB function *gradient* was applied to each of the three image channels and a single gradient image was obtained by taking the pixelwise maximum of the three individual gradient images. Finally, the mean of the resulting gradient image was adopted as an overall measure of image complexity (Braun et al., 2013).

The following five measures characterize the image color distribution.

The *Number of Colors* (NC) represents the number of distinct colors in the RGB image. For each image this number was obtained as the size of the color map resulting from the application of the MATLAB *rgb2ind* function (with the minimum variance quantization and dithering options) to each original RGB image.

*Colorfulness* is the sensation that an image appears to be more or less chromatic. Local colorfulness has been defined as a linear combination of the mean and standard deviation of the local chrominance values in color opponent space (Hasler and Süsstrunk, 2003). Note that colorfulness is not strictly related to the numbers of colors: an image can be more colorful even when its contains less different colors (Palus, 2005). A global image Colorfulness (CF) metric was computed as the mean value of the local colorfulness over a set of subwindows covering the entire image support (Hasler and Süsstrunk, 2003). CF varies from 0 (grayscale image) to 1 (most colorful image). CF was computed using the MATLAB code obtained from https://gist.github.com/zabela/8539116.

The proportional contribution of the Red (R), Green (G), and Blue (B) color channels was computed from the transparent PNG images to characterize the overall color of the (food or non-food) items (as in Blechert et al., 2014b).

Finally, the *Fraction* of the *Plate Covered* (FPC) by the (food or non-food) item on the plate was calculated for each image as follows. The total number of pixels over the area of the plate was calculated as the number of non-zero pixels in binary mask image of the empty plate. The total number of pixels covered by the item on the plate was calculated as the number of non-zero pixels in the binary image resulting after thresholding and binarization of the corresponding transparent PNG version of the image (note that this operation yields a binary mask of the area actually covered by the item). The FPC was then obtained as the ratio of both these numbers (i.e., the number of pixels representing the item divided by the number of pixels representing the plate). Note that the FPC can also be adopted as a measure for stimulus size (see Foroni et al., 2013) since the plate has the same size in all images.

### CROCUFID Availability and Use

The CROCUFID database is publicly available from the Open Science Framework repository (OSF) at https://osf.io/5jtqx with doi: 10.17605/OSF.IO/5JTQX under the CC-By Attribution 4.0 International license. Use is only allowed after complying with the following two conditions: (1) a credit line in publications and presentations reading: “*Standardized images were taken from the CROCUFID database available from the OSF repository at https://osf.io/5jtqx,”* and (2) a citation to the current article in any publication.

The database includes: the CROCUFID pictures; contact sheets providing a concise overview of all images in the dataset; documents (both in Adobe Acrobat and in Powerpoint) containing screen shots of the entire observer validation experiment; an SPSS file with the raw observer data (each participant’s code and demographic data, followed by valence, arousal, perceived healthiness and desire-to-eat ratings); an Excel file listing for each image some classifiers, the computational measures, and the mean and standard deviations of the observer ratings for the food images. The full-scene images (showing the plated items on a gray background) in the CROCUFID database are stored in JPEG format, while the item-only images (isolated items displayed on a transparent background) are in PNG format. The original RAW images and the calibration images showing the X-Rite ColorChecker Passport are available (in CR2 or DNG format) from the authors on request. The CROCUFID image database complements the F4H image collection^20^ since both were registered using the same protocol (Charbonnier et al., 2016).

## Cross-Cultural Evaluation Study

In our global economy cross-cultural research is becoming increasingly relevant in sensory and consumer science (Meiselman, 2013). Culture is one of the main factors determining human food-related behavior (Rozin, 1988). Internet-based research provides almost instantaneous world-wide access to large consumer samples, enabling researchers to conduct cross-cultural studies at relatively low-cost and in short time frames (Slater and Yani-de-Soriano, 2010; Ares, 2018). Online cross-cultural studies using images of food have for instance successfully been performed to study the effects of spatial arrangement and color composition of meals on food preference (Zampollo et al., 2012). Also, we recently used CROCUFID images in an online cross-cultural validation study of a new affective self-report tool (Kaneko et al., 2018). The item-only images in CROCUFID enable cross-cultural (online) HCI studies on the effects of environment and context on food experience (see previous section). The CROCUFID food image database is a useful tool for food-related cross-cultural research since it contains food pictures from different cuisines. Therefore, we conducted a first cross-cultural study as outlined below.

### Methods

#### Participants

Three groups completed an online anonymous survey to provide normative data for the food images in CROCUFID. The total number of participants was 805.

Two groups consisted of English speaking participants that were recruited through the Prolific survey site^21^. The text of the experiment posted on Prolific was in English. The first group (UK) comprised 266 participants from the UK. The second group (US) comprised 275 US participants. The third group (JP) consisted of Japanese speaking participants that were recruited through the Crowdworks survey site^22^. The text of the experiment was translated into Japanese for this sample. The validity of the translation was checked using the back-translation technique (Sperber, 2004). The third group comprised 264 participants. Exclusion criteria for all participants were color blindness and age (younger than 18 or older than 70).

All participants signed an informed consent form before taking part in the study and received a small financial compensation after completing the study. The experimental protocol was reviewed and approved by the TNO Ethics Committee (Ethical Approval Ref: 2017-016-EM) and was in accordance with the Helsinki Declaration of 1975, as revised in 2013 (World Medical Association, 2013).

#### Stimuli

The compilation of the CROCUFID database continued in parallel during this entire study. For the observer validation experiments reported in this study we used only the 479 food images that were already available at the start of the experiments. The remaining 361 images in CROCUFID were registered during and after the observer experiments. In the present study the stimuli were classified into four categories: a Universal food category (consisting of universally well-known fruits and vegetables etc.: *N* = 110), a typical Western food category (consisting of sandwiches, cookies, cheeses, cakes etc.: *N* = 119), a typical Asian food category (consisting of sushi, sashimi, rice-bowl, noodles etc.: *N* = 209) and an Unappealing food category (consisting of unfamiliar, rotten, molded or contaminated food: *N* = 41). Unfamiliar food was classified as unappealing since people typically evaluate (e.g., liking and willingness to try) novel foods more negative compared to familiar foods (Raudenbush and Frank, 1999).

#### Demographics and State Variables

Participants were asked to report their personal characteristics. They provided gender, age, height, weight, nationality and eating habits, such as dieting and food allergies. Participants were not requested to refrain from eating prior to taking part in the experiment, but their psychophysiological state was registered by asking them how hungry and thirsty they were feeling at the time of the experiment and how much time had passed since their last food consumption. Their state of hunger and thirst were measured with visual analog scales (VAS) labeled from “*very hungry/thirsty*” to “*not hungry/thirsty at all*.” The VAS was displayed as a solid horizontal bar, and participants responded by clicking with the mouse on the appropriate location of the line. Responses were analyzed by converting distances along the bar to a scale ranging from 0 to 100 although this was not explicitly displayed to the participants. BMI was also calculated based on participants’ reported height and weight.

#### Subjective Image Measures

For all images, valence, arousal, perceived healthiness and desire-to-eat were measured using a VAS ranging from 0 to 100 (again, this was not explicitly displayed to the participants). This is a valid method to measure food elicited affective responses (Flint et al., 2000) that has previously been used to validate food image databases (Foroni et al., 2013; Blechert et al., 2014b). Valence expresses the pleasantness of an image. The question was: “*How pleasant is the item presented in the image*?” and the extremes of the scale were labeled as “*Very unpleasant*” (0) and “*Very pleasant*” (100). The instructions for the participants explained the concept of valence by presenting some related terms for both “*Very unpleasant*” (bad, disliked, disgusting, unsatisfied, and annoyed) and “*Very pleasant*” (good, liked, delicious, satisfied, and pleased). Arousal measures the excitement (or intensity) that was experienced while viewing a food picture. The question was: “*How arousing is the item presented in the image*?” and the extremes of the scale were labeled as “*Not at all*” and “*Extremely*.” The instructions for the participants explained the concept of arousal by presenting some related terms for both the lower (calming and relaxing) and upper (stimulating and energizing) ends of the scale. Perceived healthiness was assessed with the question: “*How healthy do you think the item presented in this image is*?” and the extremes of the scale were labeled as “*Very unhealthy*” and “*Very healthy.”* Desire-to-eat was assessed with the question: “*How much would you like to eat this food right now if it was in front of you*?” and the extremes of the scale were labeled as “*Not at all*” and “*Extremely*.” The degree of recognition of the food images was also assessed by asking the question: “*Have you ever eaten the product in this image*?” that could be rated on a trichotomous scale: “*Yes*”, “*No, but I know what it is (Know)*” or “*No, I’ve never seen it before (No)*.”

#### Analysis

Normative observer data for the food images in CROCUFID were obtained through an anonymous online survey. As participants could not be expected to reliably rate all 479 food images, each participant rated only a subset of 60 images to avoid fatigue and early dropout. On average, each image was rated by approximately 100 participants including all participants from three different countries (United Kingdom, United States, and Japan).

Data was collected using Perl scripts^23^ and analyzed with IBM SPSS Statistics 23^24^. Exploratory analyses were conducted between all demographics (i.e., age, gender, nationality, height, weight, diet, and allergies), state variables (hunger, thirstiness, and time since last food intake) and subjective self-report ratings (valence, arousal, perceived healthiness, and desire-to-eat) of four classified food image categories (Universal foods, Unappealing foods, typical Western foods, and typical Asian foods) from three different nationalities (United Kingdom, United States, and Japan). In addition, the degree of recognition of four different food image categories was calculated from UK, US, and JP participants. Participants who answered “*Yes*” or “*No, but I know what it is (Know)*” were categorized as ‘recognizing’ participants, and participants who answered “*No, I’ve never seen it before*” were categorized as ‘non-recognizing’ participants.

Intraclass correlation coefficient (ICC) estimates and their 95% confident intervals were calculated based on a mean-rating (*k* = 3), absolute-agreement, 2-way mixed-effects model (Shrout and Fleiss, 1979; Koo and Li, 2016). ICC values less than 0.50 are indicative of poor reliability, values between 0.50 and 0.75 indicate moderate reliability, values between 0.75 and 0.90 indicate good reliability, while values greater than 0.90 indicate excellent reliability (Koo and Li, 2016).

#### Procedures

Participants took part in an anonymous online survey. Although internet surveys typically provide less control over the experimental conditions, they typically yield similar results as lab studies (e.g., Gosling et al., 2004; Woods et al., 2015; Majima et al., 2017) while they limiting several disadvantages associated with central location studies.

The experiment was programmed in the Java script language, and the survey itself was hosted on a web server. The time stamps of the different events (onset stimulus presentation, response clicks) and the display size and operating system of the participants were logged. This enabled us to check that participants did indeed view the stimuli on larger displays and not on mobile devices with low resolution screens. The resolution of the devices used by the participants in this study varied between 1280 × 720 and 3440 × 1440 (the average resolution was 1538 pixels × 904 pixels across participants, with standard deviations of 330 pixels × 165 pixels). We could not verify if the browser window was indeed maximized.

The survey commenced by presenting general information about the experiment and thanking participants for their interest. Then, the participants were informed that they would see 60 different food images during the experiment, and they were instructed to rate their first impression of each image without worrying about calories. It was emphasized that there were no correct or incorrect answers, and that it was important to respond seriously and intuitively. Subsequently, the participants signed an informed consent by clicking “*I agree to participate in this study*,” affirming that they were at least 18 years old and voluntarily participated in the study. The survey then continued with an assessment of the demographics and the current physical state of the participants.

Next, the participants were shown the VAS response tools together with an explanation how these could be used to report their ratings (valence, arousal, perceived healthiness, and desire to eat) for each image. Then, they performed two practice trials to further familiarize them with the use of the VAS tools. Immediately after these practice trials, the actual experiment started. During the actual experiment the participants rated 60 pseudo-randomly selected food images. The selection procedure was such that 14 images were randomly selected from the 110 Universal images, 5 from the 41 Unappealing/spoiled images, 26 from the 209 typical Asian images, and 15 from the 119 typical Western images. This selection procedure ensured that all images were rated by approximately the same number of participants and all participants viewed the same number of images from each category. The percentage of images from each category that each participant actually viewed was equal to the percentage of that particular category in the total stimulus set.

**FIGURE 5:**
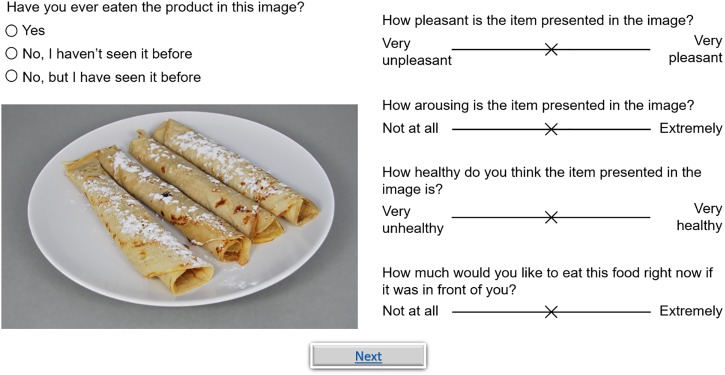
Screenshot of the display during the image rating task.

On each trial the screen displayed a food image together with the recognizability question and the four VAS tools to rate valence, arousal, perceived healthiness and desire-to-eat (for a screenshot of the experiment see Figure 5). Since this experiment was an online study with a (small) financial compensation and without any personal contact or interaction with the participants, there might be issues with motivation and seriousness. Therefore, the experiment concluded with a validated seriousness check (Aust et al., 2013) asking participants the question: “*It would be very helpful if you could tell us at this point whether you have taken part seriously, so that we can use your answers for our scientific analysis, or whether you were just clicking through to take a look at the survey?*”. Participants could select one of two answers: “*I have taken part seriously*” or “*I have just clicked through, please throw my data away*.” As an extra incentive, the following sentence was added to the instructions that were presented at the start of the experiment: “*It’s important for us that you are motivated and answer all questions seriously*.” After completing the experiment, participants received a small financial compensation and were thanked for their participation. The whole session lasted about 20 min.

### Results

There were no participants that (1) completed the study in a time span that was evidently too short in comparison to the estimated total survey duration (suggesting that they did not participate seriously) or that (2) responded negatively to the question about their seriousness were removed from further analysis. Hence, all data collected were included in the analysis.

#### Demographic Information

The average age, hunger, thirst, time since last food intake and BMI (weight in kilograms divided by height in meters squared), and the number of each gender for the three different participant groups (UK, US, and JP) is summarized in Table 3. There was a significant difference between the average age of the UK and US participants. The average time since last food-intake was significantly shorter for JP participants than for UK and US participants, which is reflected in the result that JP participants were significantly less hungry than UK and US participants. There was no significant difference between three nationality groups regarding thirst.

**Table 3:** The summary of participants’ demographics from three different nationalities (United Kingdom, United States, and Japan).

	United Kingdom	United States	Japan
Number of participants	266	275	264
Age	36.68 (±11.26)	33.04 (±11.38)	35.17 (±8.95)
Gender male(female)	83 (183)	141 (134)	101 (163)
Hunger	42.04 (±27.63)	41.34 (±26.85)	31.52 (±25.55)
Thirst	45.96 (±24.39)	44.54 (±24.13)	42.63 (±22.31)
Time since last food-intake (hr.)	4.20 (±4.59)	4.97 (±4.66)	3.40 (±2.33)
BMI	28.94 (±12.54)	27.27 (±9.37)	21.47 (±3.18)


#### Data Reliability

**FIGURE 6:**
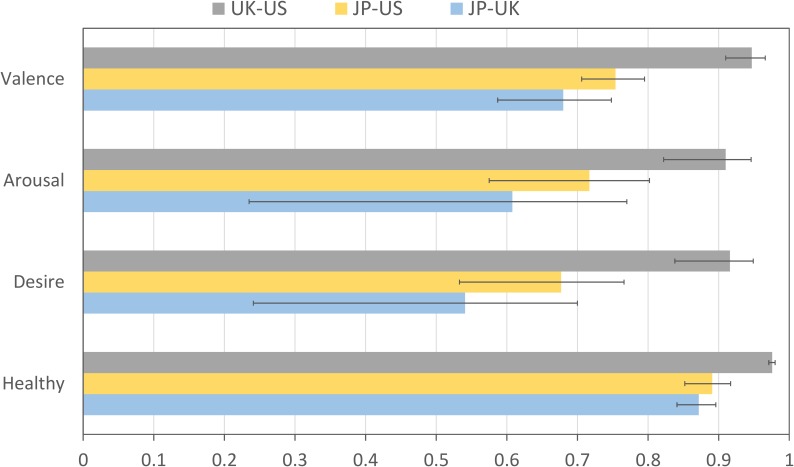
Intraclass correlation between the mean subjective (valence, arousal, desire-to-eat and perceived healthiness) ratings for each of the three different nations (United Kingdom, United States, and Japan). Error bars represent the 95% confidence intervals.

To quantify the agreement between the mean subjective image ratings provided by participants from the three different countries (Japan, United Kingdom, and United States), we computed the intraclass correlation coefficient (ICC) between each pair of countries, for each of the four subjective ratings (valence, arousal, desire-to-eat, and perceived healthiness). As shown in Figure 6, there is excellent agreement (all ICC values are larger than 0.90) between the UK and US groups for each of the four subjective ratings. The JP group shows moderate (between 0.50 and 0.75) to good (between 0.75 and 0.90) agreement with the UK and US groups. Overall, there appears to be a trend that the JP group agrees more with the US group than with the UK group.

#### Validation Ratings

For all 479 food images, we computed the average rating scores of valence, arousal, perceived healthiness, and desire-to-eat, both over all groups and within groups (UK, US, and JP). The results are provided as additional material with this paper. The food images were categorized in four different categories: Universal food (food that is globally available), Unappealing food (i.e., unfamiliar, rotten, molded, or contaminated food), typical Western food, and typical Asian food. For all three groups tested, the rating scores for Universal food images are higher than those for Unappealing food images.

##### Degree of recognition on food images across groups

**FIGURE 7:**
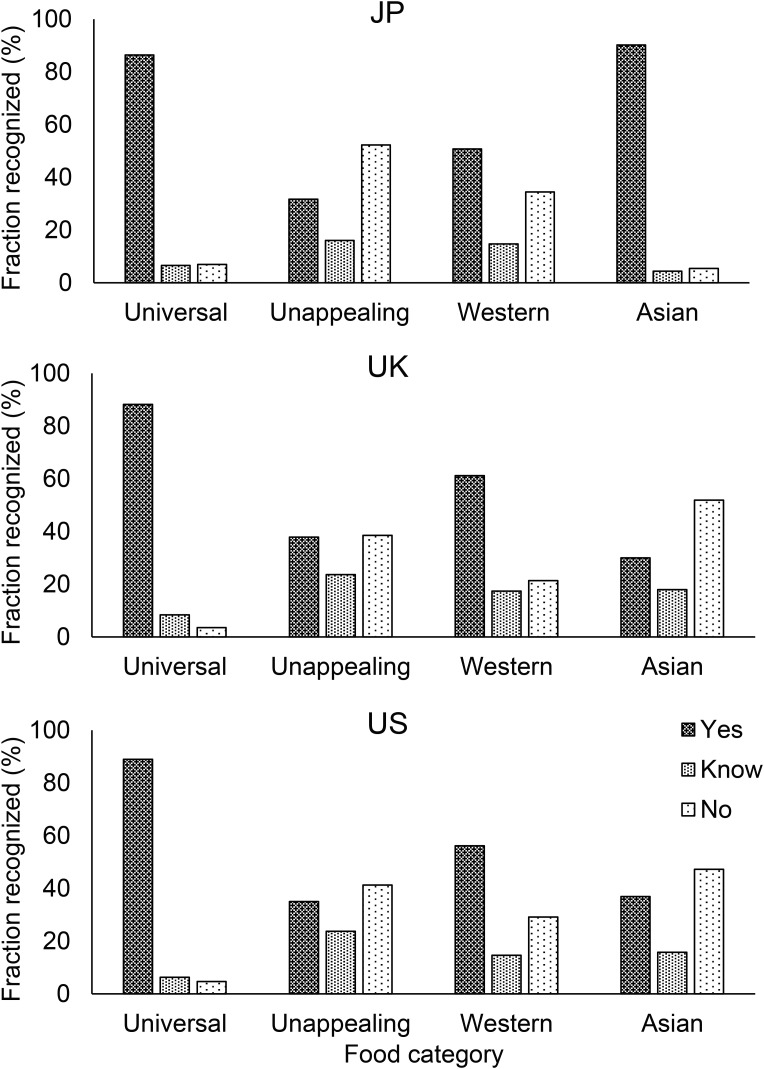
The average degree of recognition of four categories of food images rated by JP participants, UK participants, and US participants.

We also evaluated the degree of recognition for each of the four categorized food image groups across the three different groups (UK, US, and JP). Figure 7 shows for each population the average percentage who answered “*Yes*,” “*No, but I know what it is (Know)*,” and “*No, I’ve never seen it before (No)*” for each food image group. Over all three populations, 94.9% of participants recognized Universal food images on average (separately 96.6% of UK, 95.3% of US, and 93.0% of JP). UK and US participants recognized significantly more Western than Asian food images. JP participants recognized most of the Asian food images (94.6%), while only 48.0% of UK and 52.7% of US participants recognized them. There was a significant difference in the degree of recognition of Asian food images between US and JP participants, and between UK and JP participants. US participants recognized significantly more Asian images than UK participants.

##### Comparison of rating scores on Asian and Western food categories across groups (valence, arousal, perceived healthiness, and desire to eat)

**FIGURE 8:**
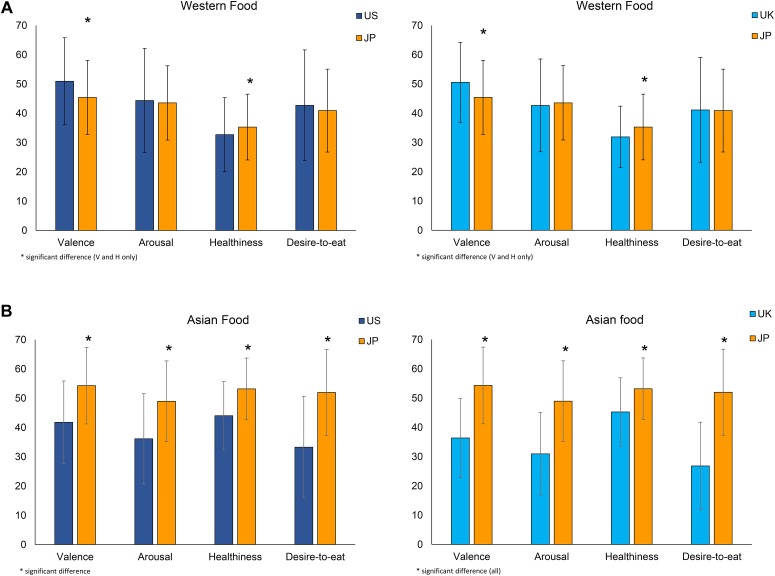
The comparison of the average rated scores of valence, arousal, healthiness and desire-to-eat on Western **(A)** and Asian **(B)** food category between UK and JP and between US and JP participants. ^∗^Indicates a significant difference between groups.

Next, we compared the average rating scores of valence, arousal, healthiness and desire-to-eat for Western (Figure 8A) and Asian (Figure 8B) food categories between UK and JP and between US and JP participants. US and UK participants rated Western food significantly higher on valence than JP participants. There was no significant difference between the rating scores on arousal and desire-to-eat for Western food and for each of the groups tested (US and UK and JP). JP participants rated Asian food images significantly higher than UK and US participants on all items.

#### Computational Image Measures

The main purpose of providing computational image characteristics with CROCUFID is to allow the user to select CROCUFID images based on their physical properties. Since the selected features are known (or *a priori* likely) to influence image appraisal we also computed the correlation between the computational metrics and the mean observer ratings. Table 4 lists the significant Pearson correlations between the mean observer ratings (overall and for each of the three groups individually) and each of the computational image measures.

**Table 4:** Pearson correlations between the mean observer ratings (overall and for each of the three groups individually) and the computational image measures.

		Computational measure																					
	
	Texture							Complexity								Color						
	
Mean observer rating		S	P	C	E	H	SS	AN	CRjpeg	CRgif	FC	SE	NPO	MIGh	MIGs	MIGv	MGS	NC	CF	R	G	B
Valence	All	**0.238**^∗∗^			-0.153^∗∗^		**0.201**^∗∗^	-0.151^∗∗^	0.137^∗∗^	0.133^∗∗^	0.177^∗∗^		0.109^∗^					**0.268^∗∗^**	**0.353^∗∗^**			
	UK	**0.264**^∗∗^			-0.179^∗∗^	-0.095^∗^	**0.300**^∗∗^	-**0.329**^∗∗^	**0.243**^∗∗^	**0.226^∗∗^**	**0.295^∗∗^**	0.099^∗^	0.130^∗∗^		0.152^∗∗^	0.122^∗∗^	0.134^∗∗^	**0.290^∗∗^**	**0.406^∗∗^**			
	US	**0.273**^∗∗^			-0.170^∗∗^		**0.256**^∗∗^	-**0.253**^∗∗^	**0.200**^∗∗^	0.194^∗∗^	0.**254**^∗∗^		0.125^∗∗^		0.119^∗∗^		0.103^∗^	**0.278^∗∗^**	**0.366^∗∗^**			
	JP		-0.124^∗∗^	-0.133^∗∗^		.118^∗∗^		**0.242**^∗∗^	-0.117^∗^	-0.100^∗^	-0.124^∗∗^	-0.151^∗∗^				-0.149^∗∗^	-0.100^∗^	0.137^∗∗^	0.148^∗∗^			

Arousal	All	**0**.**266**^∗∗^			-0.190^∗∗^		**0.219**^∗∗^	-0.187^∗∗^	0.190^∗∗^	0.170^∗∗^	**0.204^∗∗^**		0.158^∗∗^		0.144^∗∗^		0.122^∗∗^	**0.345^∗∗^**	**0.375^∗∗^**			
	UK	**0.260^∗∗^**			-0.189^∗∗^	-0.119^∗∗^	**0.294**^∗∗^	-**0.328**^∗∗^	**0.258^∗∗^**	**0.230^∗∗^**	**0.288^∗∗^**	0.120^∗∗^	0.167^∗∗^		0.179^∗∗^	0.148^∗∗^	0.159^∗∗^	**0.328^∗∗^**	**0.402^∗∗^**			
	US	**0.295**^∗∗^			-0.199^∗∗^	-0.112^∗^	**0.257**^∗∗^	-**0.269**^∗∗^	**0.236^∗∗^**	**0.214^∗∗^**	**0.262^∗∗^**	0.109^∗^	0.170^∗∗^		0.168^∗∗^	0.118^∗∗^	0.153^∗∗^	**0.344^∗∗^**	**0.365^∗∗^**			
	JP	0.133^∗∗^			-0.109^∗^			0.161^∗∗^										**0.238^∗∗^**	**0.207^∗∗^**			

Healthiness	All	**0.200**^∗∗^	-0.092^∗^				0.148^∗∗^						**0.312**^∗∗^					**0.204**^∗∗^	0.134^∗∗^	-0.119^∗∗^	0.148^∗∗^	
	UK	**0.239**^∗∗^					0.189^∗∗^						**0.317**^∗∗^					**0.243**^∗∗^	0.167^∗∗^	-0.125^∗∗^	0.150^∗∗^	
	US	**0.233**^∗∗^					**0.208**^∗∗^						**0.314**^∗∗^					**0.223**^∗∗^	0.172^∗∗^	-0.115^∗^	0.155^∗∗^	
	JP		-0.136^∗∗^	-0.124^∗∗^		0.160^∗∗^		0.189^∗∗^	-0.172^∗∗^	-0.132^∗∗^	**-0.200**^∗∗^		**0.255**^∗∗^		-0.101^∗^	-0.170^∗∗^	-0.120^∗∗^	0.099^∗^		-0.098^∗^	0.113^∗^	

Desire-to-eat	All	**0.244**^∗∗^			-0.153^∗∗^		**0.214**^∗∗^	-0.161^∗∗^	0.170^∗∗^	0.167^∗∗^	**0.209**^∗∗^		0.105^∗^		0.109^∗^			**0.244**^∗∗^	**0.353**^∗∗^			
	UK	**0.275**^∗∗^		0.097^∗^	-0.184^∗∗^	-0.141^∗∗^	**0.334**^∗∗^	**-0.382**^∗∗^	**0.300**^∗∗^	**0.283**^∗∗^	**0.352**^∗∗^	0.144^∗∗^	0.122^∗∗^		0.186^∗∗^	0.179^∗∗^	0.176^∗∗^	**0.261^∗∗^**	**0.410^∗∗^**			
	US	**0.280**^∗∗^			-0.188^∗∗^	-0.111^∗^	**0.267**^∗∗^	**-0.282**^∗∗^	**0.249**^∗∗^	**0.237**^∗∗^	**0.300**^∗∗^	-0.103^∗^	0.123^∗∗^		0.157^∗∗^	0.123^∗∗^	0.143^∗∗^	**0.259^∗∗^**	**0.374^∗∗^**			
	JP		-0.125^∗∗^	-0.137^∗∗^		0.124^∗∗^		**0.275**^∗∗^	-0.128^∗∗^	-0.107^∗^	-0.132^∗∗^	-0.158^∗∗^				-0.155^∗∗^	-0.109^∗^	0.107^∗^	0.119^∗∗^			


**^∗^Correlation is significant at the 0.05 level (2-tailed). ^∗∗^Correlation is significant at the 0.01 level (2-tailed). Values above 0.2 are printed in bold. Only significant correlations are reported.**

Although most correlations are small (between 0 and 0.3), there is still an appreciable number of medium sized correlations (between 0.3 and 0.5), particularly for the texture metrics AN and SS, and the color metrics NC and CF.

##### Image texture

The image texture metrics S, SS, and AN appear to have the largest absolute overall correlation with mean observer ratings for valence, arousal and desire-to-eat. For the UK and US groups, S and SS are positively correlated with all four of the mean observer ratings, meaning that for these groups valence, arousal, perceived healthiness and desire-to-eat all increase with increasing randomness (S) and self-similarity (SS) of the food items. For the JP group, S is only weakly positively correlated with arousal, while SS is not significantly correlated with any of the mean ratings, suggesting that randomness and self-similarity do not strongly affect the appraisal of food items for this group. AN (which is inversely related to disorder) correlates negatively with each of the four mean ratings for the UK and US groups, while it correlates positively with each of the mean ratings for the JP group. This means that increasing variations in local orientations over the area of a food item decreases each of the four mean ratings for the UK and US groups, while it increases these ratings for the JP group.

##### Complexity

The complexity metrics CR_jpeg_, CR_gif_, and FC appear to have the largest absolute positive overall correlation with mean observer ratings for valence, arousal and desire-to-eat. This correlation is positive for the UK and US groups, but negative for the JP group, meaning that perceived valence, arousal and desire-to-eat increase with increasing complexity for the UK and US groups, while they decrease with complexity for the JP group. NPO is the only complexity measure that systematically correlates positively with all four measures for all three groups. This means that all groups rate perceived healthiness higher when food items are composed of a larger number of distinguishable components.

##### Color

The color metrics NC and CF are positively associated with all four measures for all three groups. However, compared to the UK and US groups, this correlation is quite weak for the JP group. Hence, it seems that valence, arousal, perceived healthiness and desire-to-eat all increase with increasing number of colors and colorfulness of the food items, but more so for the UK and US groups than for the JP group.

##### Cultural differences

The results listed in Table 4 show that the relation between the mean subjective image ratings and each of the most predictive image metrics differs consistently between the JP groups and the UK and US groups: the variation of the mean (valence, arousal, desire-to-eat, and perceived healthiness) ratings of the JP group with each of the texture, complexity and color metrics is consistently smaller or even opposite to those of the UK and US groups. A graphical overview of these results is provided in the Supplementary Material with this article.

Arousal, valence, and desire-to-eat consistently increase with increasing structural food complexity (i.e., with increasing values of the texture, complexity and color metrics) for the UK and US groups (see Table 4). On these three subjective measures, the JP group shows an opposite behavior to the UK and US groups for the texture measure AN and for each of the three complexity measures CR_jpeg_, CR_gif_, and FC. Like the UK and US groups, the mean arousal ratings by the JP group also increase with increasing food item colorfulness or number of colors, though less strongly. Perceived healthiness increases with the number of colors (NC), distinguishable components (NPO) and randomness (S) of the food items, though less strongly for the JP group than for the UK and US groups.

## Discussion

The CROCUFID database contains high-quality images that are registered according to standardized protocol, covering the full valence and arousal space. CROCUFID includes multiple cuisines and unfamiliar foods and provides normative and demographic data. The database is hosted in the OSF repository^25^ and is freely available under the CC-By Attribution 4.0 International license. We plan to extend this image collection with food images from different cuisines in addition to the Western and Asian foods that are currently included. Also, the CROCUFID image collection can easily be extended by linking it to similar data sets. Researchers who are interested in contributing to this effort are kindly invited to provide the authors with a link to their image dataset. The images should preferably be documented and registered according to the protocol presented by Charbonnier et al. (2016).

As expected, the present results of our cross-cultural study show that the JP group recognized significantly more Asian food compared to UK and US groups, while the UK and US groups recognized significantly more Western food than the JP groups, suggesting that Asian food images seem to have a strong cultural “bias”: for Western group (US and UK) it is hard to distinguish different food images. As also expected, JP group who has a higher degree of recognition of Asian food images rated significantly higher scores of valence, arousal, healthiness, and desire-to-eat than Western groups (US and UK). Regarding the rating scores on Western food images, Western groups (US and UK) rated significantly higher scores of valence than JP group. There was no significant difference in the rating scores of arousal and desire-to-eat between the western group and the JP group. The results of valence ratings on Asian and Western food by the JP group and the Western groups (UK and US) agree with the previous findings of Knaapila et al. (2017) that the familiarity of spice odors the participants have was positively associated with pleasantness. Fenko et al. (2015) also found similar results using soy products, where familiar products were evaluated more positively than unfamiliar products with German participants.

Our current result that valence, arousal, desire-to-eat and perceived healthiness are positively associated with all texture measures (spatial disorder) for the UK and US groups and less strongly with some of them for the JP group agrees with the previous finding of Zampollo et al. (2012) that participants from the United States and Italy tend to prefer disorganized food presentations, while Japanese participants do not have a significant preference.

The present result that valence, arousal, desire-to-eat and perceived healthiness are positively associated with all measures of complexity for the UK and US groups agrees with the results of many previous studies that reported a positive association between visual complexity and affective (i.e., valence and arousal) ratings: complex stimuli are typically perceived as more pleasant and more arousing than less complex ones (for an overview see Madan et al., 2018). However, the JP group shows the opposite behavior: mean valence, arousal and desire-to-eat ratings are all negatively associated with complexity for this group.

Our finding that the number of identifiable components (NPO) is positively associated with perceived healthiness agrees with previous observations that (1) people prefer servings composed of multiple pieces to single-piece ones (Wadhera and Capaldi-Phillips, 2014), and (2) food cut in smaller pieces is preferred to larger chunks (Wadhera and Capaldi-Phillips, 2014), probably because segregating food into multiple components increases the perceived variety of the foods and is therefore perceived as more rewarding (Levitsky et al., 2012). It has also been suggested that perceived variety (e.g., through multiple colors or components) may be preferred in food since it typically delays the sensory-specific satiety (Wadhera and Capaldi-Phillips, 2014).

Our finding that a higher intensity of the green color channel is positively associated with perceived healthiness, while a higher intensity of the red color channel is negatively correlated with healthiness, agrees with previous reports that a higher intensity of the green channel was positively associated with lower concentrations of protein, fat and carbohydrates Blechert et al., 2014b) and with a lower (perceived) number of calories (Blechert et al., 2014b; Foroni et al., 2016), while a higher intensity of the red channel was positively associated with energy density (Foroni et al., 2016).

Finally, the result that the number of colors (NC) and colorfulness (CF) are positively associated with that the mean valence, arousal, desire-to-eat and perceived healthiness ratings agrees with previous reports in the literature that multicolored food is rated higher in attractiveness (Zellner et al., 2010) and pleasantness (Rolls et al., 1982) than single-colored food, and that colorfulness is typically positively associated with healthiness (König and Renner, 2018).

This study also has some limitations. First, since the validation rating internet survey was conducted during the construction of CROCUFID, not all food (non-food) images have been rated yet. Second, since all participants were randomly collected from the UK, US, and Japan, there is no information about their exact region of origin. Hence, additional validation survey is needed to complete the database and specify cross-cultural effects within each country.

In conclusion, CROCUFID currently contains 840 food images (675 food images and 165 non-food images), from different (Western and Asian) cuisines and with different degrees of valence. CROCUFID also includes computational image characteristics regarding visual texture, complexity, and colorfulness, that may be used to select images for different applications (e.g., food research, automatic image interpretation, and VR/AR applications). The accompanying validation data are derived from a total of 805 participants (ranging in age from of 18–70) with different cultural backgrounds (UK, US, and JP). CROCUFID may also be used to conduct (neuro)physiological studies because all items are shown in the same background context and are also available as transparent overlays. Currently many different food cuisines are still lacking from CROCUFID. We hope to extend the collection with images of food from other cuisines. Researchers from different parts of the world are kindly invited to contribute to this effort by creating similar image sets that can be linked to this collection, so that CROCUFID will grow into a truly multicultural food database.

## Author Contributions

AT, IdK, DK, A-MB, VK, and JvE conceived and designed the study. IdK and SU registered and compiled the image database. IdK and DK performed the online validation study. AT processed the imagery, calculated the computational image measures, and curated the data. IdK and MvS performed the statistical analysis of the results. AT, DK, and IdK wrote the manuscript. All authors contributed to manuscript revision, read and approved the submitted version.

## Conflict of Interest Statement

The authors declare that the research was conducted in the absence of any commercial or financial relationships that could be construed as a potential conflict of interest.

## Footnotes

1 https://foodcast.sissa.it/neuroscience

2 http://www.eat.sbg.ac.at/resources/food-pics

3 https://doi.org/10.5281/zenodo.10202

4 http://nutritionalneuroscience.eu

5 http://pfid.rit.albany.edu

6 http://www.murase.is.i.nagoya-u.ac.jp/nufood

7 https://sites.google.com/view/chinesefoodnet/

8 http://iplab.dmi.unict.it/UNICT-FD889

9 http://foodcam.mobi/dataset.html

10 https://www.vision.ee.ethz.ch/datasets_extra/food-101/

11 http://vireo.cs.cityu.edu.hk/VireoFood172/

12 www.ikea.com

13 http://orangemonkie.com/foldio2

14 http://xritephoto.com

15 http://www.mathworks.com

16 www.photoscissors.com

17 http://www.mit.edu/∼yzli/clutter.htm

18 http://www.mit.edu/∼{}yzli/clutter.htm

19 http://www.chenpingyu.org/projects/proto-objects.html

20 http://nutritionalneuroscience.eu

21 www.prolific.ac

22 https://crowdworks.jp

23 www.perl.com

24 www.ibm.com

25 https://osf.io/5jtqx
