# Supplementary material for: CROCUFID: A Cross-Cultural Food Image Database for Research on Food Elicited Affective Responses
Source: Front Psychol. 2019 Jan 25;10:58. doi: 10.3389/fpsyg.2019.00058 (PMC6355693; doi:10.3389/fpsyg.2019.00058)
Supplement: Supplementary file 1 [file Data_Sheet_1.PDF]

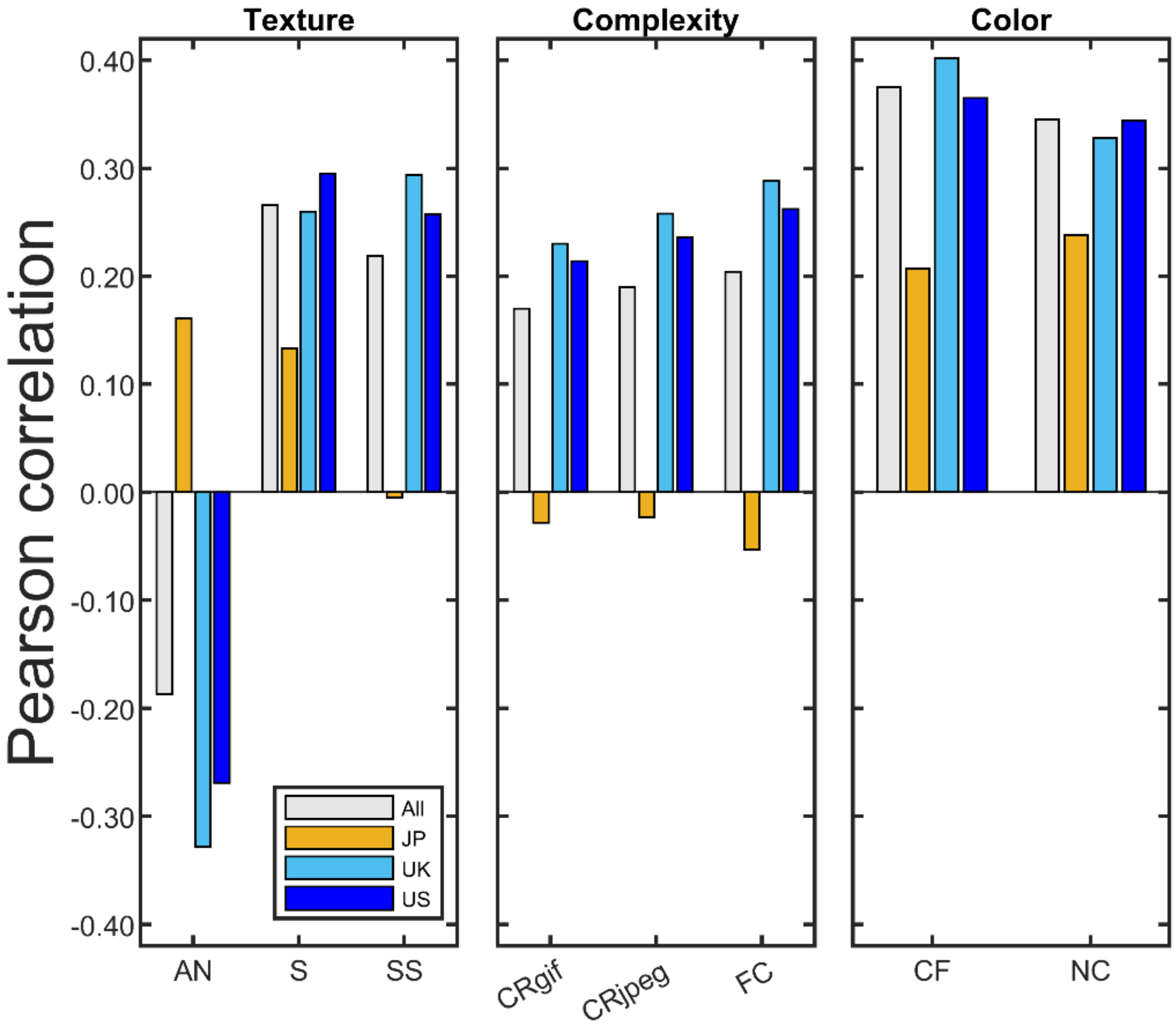

Figure S1. Pearson correlation between the mean arousal ratings for each of the three groups (UK, US and JP) and the (texture, complexity and color) image metrics with the largest and most consistent overall correlation (see Table 4).

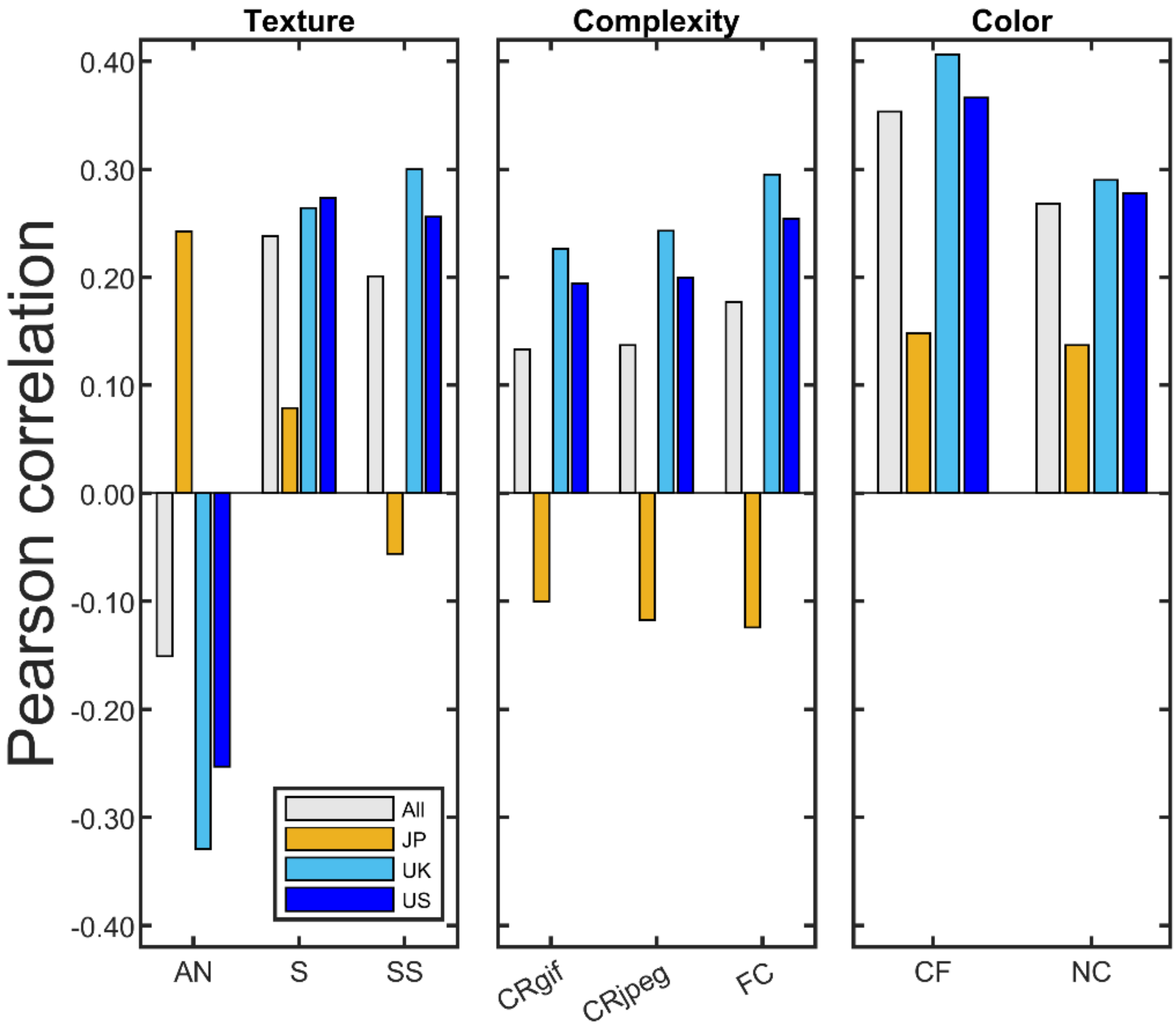

Figure S2. Pearson correlation between the mean valence ratings for each of the three groups (UK, US and JP) and the (texture, complexity and color) image metrics with the largest and most consistent overall correlation (see Table 4).

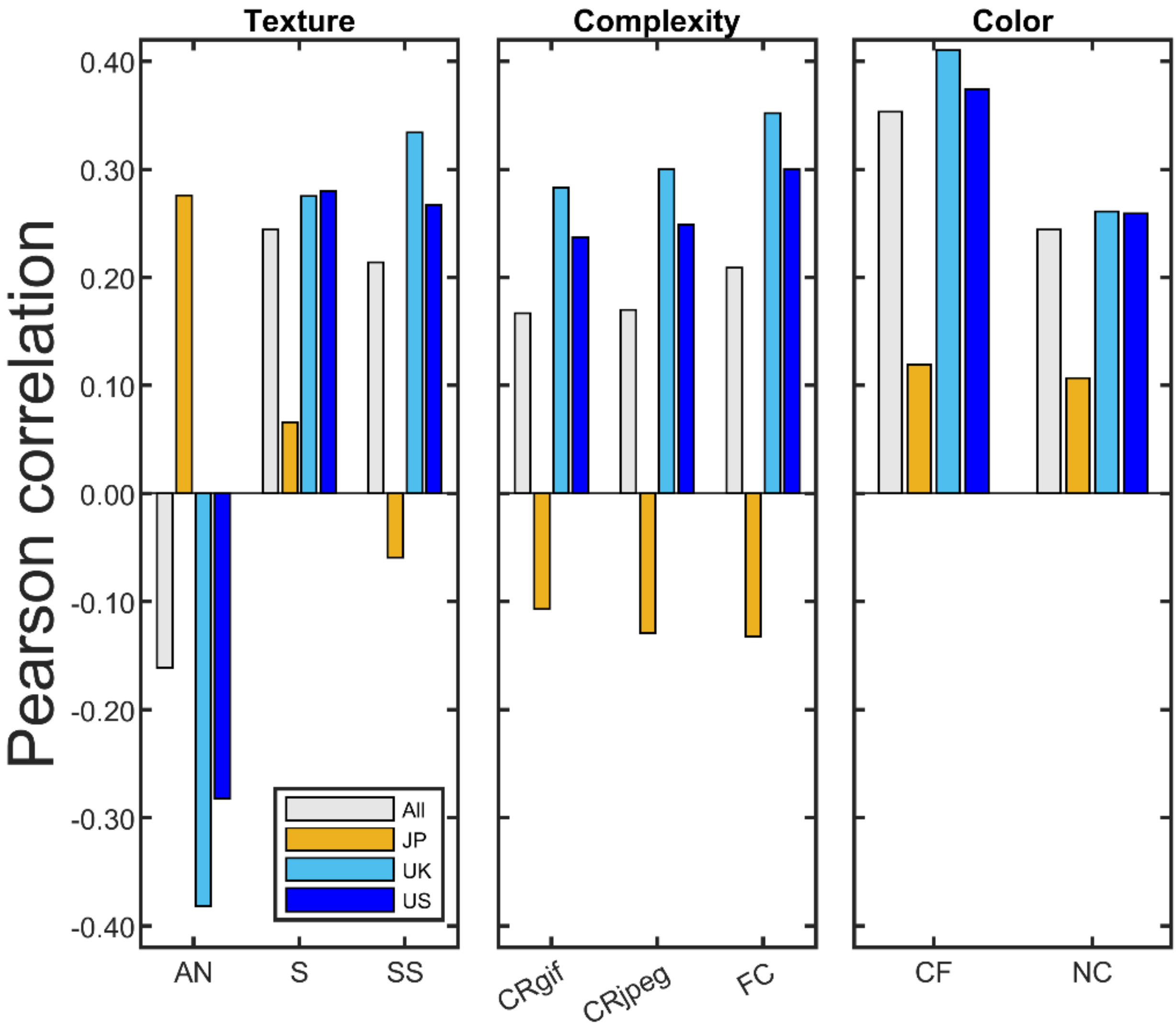

Figure S3. Pearson correlation between the mean desire-to-eat ratings for each of the three groups (UK, US and JP) and the (texture, complexity and color) image metrics with the largest and most consistent overall correlation (see Table 4).

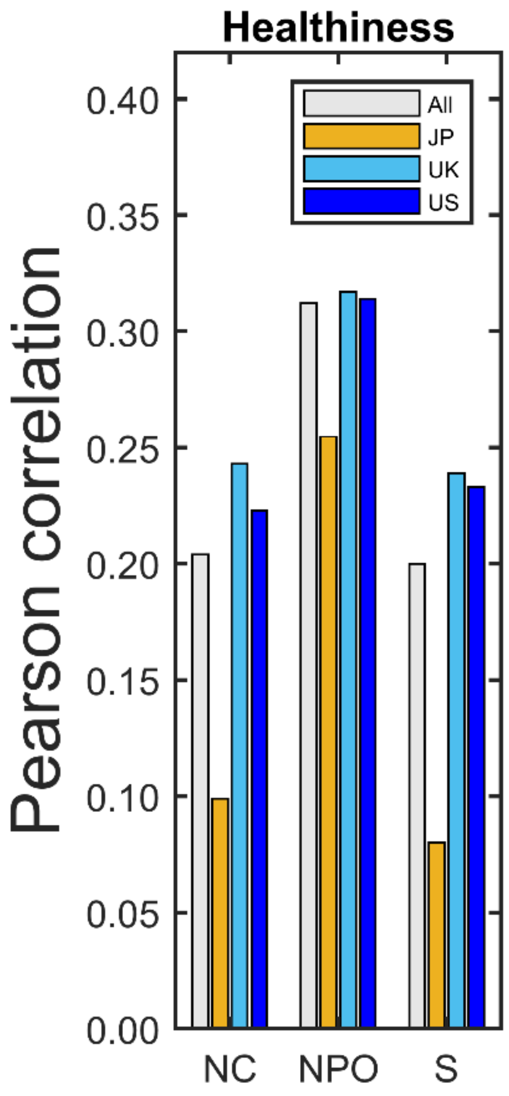

Figure S4. Pearson correlation between the mean perceive healthiness ratings for each of the three groups (UK, US and JP) and the (texture, complexity and color) image metrics with the largest and most consistent overall correlation (see Table 4).
